# Professional Competence or Personal Relationship? Research on the Influencing Mechanism on Repeated Purchase Intention of Agricultural Resources

**DOI:** 10.3390/ijerph17072278

**Published:** 2020-03-28

**Authors:** Lan Li, Gang Li, Junqi Chen

**Affiliations:** 1 School of Business Administration, Research Center of Henan Economy, Henan University of Economics and Law, Zhengzhou 450046, China; lilan@huel.edu.cn; 2 School of Management and Economics, North China University of Water Resources and Electric Power, Zhengzhou 450045, China

**Keywords:** personal relationship, professional competence, repeated purchase intention, agricultural marketing

## Abstract

Based on social exchange, rational choice, and perceptual choice theory, this paper examines the influence of professional competence, personal relationship, and their interaction on repeated purchase intention in the context of rural agricultural marketing. Adopting the survey method and hierarchical regression analysis, this study tested the hypotheses with a data set of 578 farmers from China, and assessed the robustness of the results by structural equation modelling. The results show that both personal relationship and professional competence have a significantly positive impact on repeated purchase intention while the interaction between the two has a significant negative effect on repeated purchase intention. The results expose the struggle farmers experience in choosing between emotional and rational thinking when making purchasing decisions in the urbanization and industrialization process of a rural area. The results also enrich the research on the marketing of agricultural resources and have important implications to agricultural retailers.

## 1. Introduction

Repeated purchase behavior plays a key role in maintaining a stable market share of retailers. Therefore, the research on the influencing factors of repeated purchase behavior has attracted attention from both academia and marketers. The most significant characteristic of repeated purchases as a behavior is their instability and immeasurability. Although the result of using repeated purchase intention to predict repeated purchase behavior is higher than the fact, as a conscious characterization of repeated purchase behavior, repeated purchase intention is still a relatively reliable and stable predictor. Therefore, repeated purchase intention is used to predict repeated purchase behavior by most of the extant research [1]. So far, the influencing factors of repeated purchase intention have been examined by extant research, including trust [2,3], satisfaction [4], perceived quality [5], switching cost [6], and relationship [7,8]. Except for switching cost, which has a negative effect on repeated purchase intention, the results of these studies generally show that other factors positively affect repeated purchase intention.

The personal relationship between consumers and retailers is an important influencing factor of repeated purchase intention [7,8]. This is especially true in rural areas, where people’s production and living areas are more limited compared with those in cities due to the relatively closed social circle [9,10]. With blood and geography as the initial endowment of interpersonal relationships, people maintain independent social circles in villages. An “acquaintance society” is thus formed. This relatively closed and independent traditional rural social structure makes personal relations have a more significant and direct impact on rural economic behavior, such as agricultural resource purchasing [9,11]. 

Professional competence is the perceived skills and knowledge that salespersons have when they provide services or products to customers. It involves salespersons’ understanding of the products, markets, and their performance of guiding farmers to purchase suitable agricultural resources [12]. In many countries, farmers generally have a lower level of education compared with urban residents, and have very limited knowledge or expertise in identifying differences between various types of agricultural resources [13]. Therefore, the professional competence of agricultural salespersons is essential for farmers to purchase the right agricultural resources and to protect their interests. At the same time, when rural areas go through an urbanization and industrialization process, the mobility of rural personnel increases, and rural society changes from the traditionally closed “indigenous society” to a semi-closed “post-native” society [14]. Farmers turn from affective thinking and limited acting [15] to a state of coexistence of sensibility and rationality [16,17,18]. Therefore, the salesperson’s professional competence, as a key factor affecting farmers’ agricultural production, is increasingly and rationally considered by farmers in purchasing decisions.

The existing literature has the following shortcomings. Firstly, the extant research on repeated purchase intention in the context of rural agricultural purchasing and sales needs to be improved. Previous studies on repeated purchase intention are mostly based on business-to-business marketing, consumer products retailing in cities, or online marketing, and few studies have been based in a rural context. Therefore, the question that remains unanswered is in the rural agricultural purchasing and sales context, will the factors affecting the repeated purchase intention differ due to the special circumstances of the trading environment, the participants of the transaction, or the product exchanged?

Secondly, the relationship between professional competence and repeated purchase intention in the rural agricultural transaction needs to be verified. Although research done by scholars has confirmed a positive effect of professional competence on repeated purchases in the retail industry [19,20,21], the question needed to be answered is: Is the relationship still the same in a different social context and industrial background?

Furthermore, since the personal relationship and professional competence simultaneously affect the repeated purchase intention of farmers, the effect of the interaction between the two on repeated purchase intention needs to be explored. In many countries, relations and abilities both play an important role in social life, and very often the interaction of the two makes the effects more unpredictable. Thus, the questions raised are: Do the two variables, related to emotions and rationality, respectively, have a common effect on repeated purchase intention in the context of agricultural materials transaction? If so, what is the mechanism of the influence?

Finally, regarding the transformation of rural social structure and the change of farmers’ mode of thinking in the process of industrialization and urbanization of rural areas of many countries, previous studies have only expounded from a theoretical perspective. Therefore, the questions remain unanswered are: During the transformation of the social structure and social relationship, did the farmers, as the actor of behavior, change from emotional thinking to rational thinking as theoretically proposed? What is the current state? How do farmers make purchase decisions in this state? Thus, this paper will also analyze, from the perspective of sociology, the changes in rural social relations and farmers’ mode of thinking during the process of industrialization, and urbanization of rural areas. 

The personal relationship is considered to be one of the most important mechanisms in Chinese society [22,23], and is the basis and most significant feature of business interactions in the context of Chinese culture [24]. Competition in the agricultural resources market in China has intensified in recent years. In addition, the deceitful operation of some agricultural retailers has led farmers to rely more on honest retailers for repeated purchases. Furthermore, rural Chinese areas are undergoing a significant transition through the industrialization and urbanization process. Therefore, this paper used China as an example to study repeated purchase intention in a scenario of agricultural resource transaction. 

Based on social exchange, rational selection, and perceptual selection theory, it empirically examines the effect of the personal relationship, professional competence, and their interaction on repeated purchase intention. This study aimed to supplement the research on purchase intention in agricultural marketing. It also provides a reference for rural agricultural resource retailers to attract customers, and to maintain a stable market share. At the same time, it enriches sociology studies of the changes in farmers’ thinking characteristics and its impact on their behavior in the process of rural urbanization and industrialization.

## 2. Theoretical Base

### 2.1. Personal Relationship

The existing literature interprets personal relationships as special exchange relationships, a type of asset, and a dynamic process of association. For example, Devaraj et al. regards relationships as a fact of existence, a purposeful way of behavior, and an exchange of emotions or interests [25]. They believe that the personal relationship is a dynamic and continuous process that involves the social exchange of two individuals. Rao et al. argues that the personal relationship is an exchange relationship composed of individual relationships, including the inherent characteristics of mutual responsibility and satisfaction of needs [26]. Lee and Davies claim that the personal relationship is a connection between individuals who gain a resource advantage in the transaction process [27]. The relationship between the two parties is mutually beneficial and this connection can be a bridge to achieve certain goals [27].

Ramasamy et al. defined the personal relationship as an informal social activity relationship outside the workplace [28]. Wang and Chao believe that the personal relationship refers to the blood relationship and kinship between people, and includes “symbolic” kinship, such as fellow villagers, friends, classmates, and a friend relationship other than a working relationship [29]. Personal relationships are a kind of directional relationship that is derived from a relationship and interpersonal relationship but is different from the two. Compared with a general “relationship”, it has a smaller scope; compared with an “interpersonal relationship”, it focuses more on people’s emotions with each other, rather than doing things together. It emphasizes “private” and refers to a relationship with an emotional nature other than a working relationship.

In the service industry, customers have frequent contact with corporate service personnel, and independently determine consuming behavior. The relationship between them shows strong affection and autonomy [30]. In rural society, the geographical environment is relatively closed, and people are in frequent contact with people who live nearby. In addition, the blood and kinship generated by a family clan and intermarriage in the village lead to an “acquaintance society”. The agricultural salespersons, including the owners of the agricultural retail stores, are usually the nearest villagers, and the relationship with the farmers is originally an acquaintance relationship. According to the survey results of this project, 90% of the farmers are small-scale growers with a cultivated area of less than 15 acres. They all choose to purchase agricultural materials from the agricultural retail stores in the nearby villages and towns. However, the number of agricultural retail outlets in a village is relatively small, generally no more than 10, thus the frequency of contact between farmers and agricultural salespersons is very intensive, and farmers are more familiar with and understand agricultural salespersons.

In the long history of China, relationships are not only an important part of people’s daily life but also an important social resource to them [31]. Personal relationships have a special status in Chinese culture. Popular phrases, such as “blood relationship” and “nepotism”, not only include the literal meaning of the word “relationship” but also indicate the intricate cultural origin. Influenced by traditional Chinese culture, Chinese people value the emotional component of personal relationships [32]. Rural society in China is relatively traditional. Compared with urban residents, farmers generally interact with others emotionally. Over time, farmers and agricultural salespersons will have emotional personal relationships as their familiarity increases. Based on the research context of the agricultural resources transaction, this paper integrates the definitions of Wang and Chao [30] and Ramasamy et al. [28], and refers to personal relationships as blood relationships and kinship, and as a type of “symbolic” kinship that includes fellow villagers, friends, classmates, and is a kind of friend relationship other than a work relationship, which highlights the importance of emotion.

### 2.2. Professional Competence

Professional competence refers to the level of skills and knowledge that sales staff have in providing basic services or products to customers, which is usually expressed in the form of information provided by sales staff [12]. Studies have shown that professional competence requires the combination of knowledge and competence, which is the ability to translate knowledge into strong performance [33,34,35]. There is also the definition of professional competence as an individual, which refers to an expert who possess specific skills or professions in a specific field [36]. In general, professional competence can be defined as a personal ability that uses professional knowledge to achieve extraordinary performance, such as sales planning in a specific context [37] and adaptive sales [38,39].

Some scholars have defined professional competence from the perspective of affected parties, and believe that professional competence is the perception and recognition of services or skills and knowledge provided by the other party in a dual relationship. For example, Busch and Wilson believe that professional competence is the value perception of the affected person about the affecting person’s knowledge, information, or skills in a certain area [40]. Professional competence is a source of influence, but it must be generated in a binary relationship and cannot be perceived by a third party. Shamdasani and Balakrishnan argue that professional competencies include skills and knowledge in certain aspects, which can provide the other party with a higher degree of satisfaction and trust [41]. The customer mainly judges the professional competence of the salesperson by the perceived information in the process of receiving related services.

Disregarding observations from differing perspective, these definitions of professional competence refer to the same thing, i.e., it is the level of skills and knowledge that the salesperson has when they provide the customer with basic services or products. It mainly includes knowledge of the product and the market, and the performance of the salesperson in guiding customers to buy suitable products. In rural China, in addition to farmers’ non-professional characteristics of agricultural resources [42], rural agricultural technicians are generally absent, and farmers basically do not receive necessary technical assistance from agricultural technicians. Therefore, the effectiveness of purchasing the right agricultural resources will depend to a large extent on the guidance of agricultural resource salespersons and their professional competence. This is also a key issue that farmers need to face and deal with rationally when it comes to economic benefits. Therefore, this study considers professional competence as an independent variable of the rational choice of farmers in agricultural resource purchasing.

### 2.3. Repeated Purchase Intention

Repeated purchase intention refers to the subjective intention of the customer to purchase a certain product or service again, if necessary, after using the product or receiving the service [6,43,44,45]. So far, some scholars have explored the impact of relationships on repeated purchase intention, such as relationship quality and interpersonal relationships [46]. For example, Zhang conducted a study on the online shopping experience of undergraduates and graduate students in a university in Tianjin of China [47]. The investigation empirically verified the positive impact of brand identity, brand commitment, brand loyalty, and emotional attachment commitment on repeated purchase intention. Li and Xiao examined the relationship between service quality, relationship quality, and customer repurchase intention in the context of chain drugstores, and concluded that relationship quality positively affects customer repurchase intention [48]. From the perspective of buyers, Li and Lu studied the relationship between the quality of the brand relationship and the purchase intention of buyers, and concluded that the quality of the brand relationship plays a key role in maintaining the relationship of repurchasing [49]. However, generally speaking, only a few studies have investigated the effect of “relationships” on repeated purchase intention.

## 3. Hypotheses Development

### 3.1. Personal Relationship and Repeated Purchase Intention

Personal relationships have a significant effect on maintaining customer loyalty and improving corporate performance [23]. This effect is even more pronounced in rural areas. Limited by the difficult transportation and small social network in rural areas, direct contact between people is denser and more frequent in rural areas, so it is natural to form “acquaintance relationships”. In this traditional acquaintance society, people’s behavior is embedded in relationships, and relations follow a pattern of “self-centered” ripples from the near to the far [42]. The interaction between people follows the inner emotions and acquaintance relationships, which falls in an “ethical sensibility” model [42]. In a society such as this, business activities, relationships, and feelings often come before interest exchange [32].

Some studies have found that this structure makes personal relationships in rural societies have a more direct impact on rural economic behavior [11]. Interpersonal relationships in the rural area will affect the stability of the transactional relationship between farmers and agricultural retailers and their transactional behaviors [48,50]. A good relationship means being familiar with one another and being emotionally close. With the trust that emerges from the “kinship relationship”, and the pleasant feelings farmers get from their interactions, it is easier to trigger market transactions and make them repeat their purchases [10,32,51]. In addition, in the battle for survival in the highly competitive agricultural resource market, farmers are more inclined to take care of and help their “own people”. Enterprises pay important attention to the development and maintenance of personal relationships with farmers [52]. Therefore, it is proposed that, when all else is equal:

**Hypothesis 1** **(H1).** *The personal relationship between farmers and agricultural retailers positively affects farmers’ repeated purchase intention*.

### 3.2. Professional Competence and Repeated Purchase Intention

The professional competence of sales or service personnel is the decisive factor for customers to be able to trust them [53]. Mitchell and Dacin found in a study on consumer expertise that those salespersons who are considered to have expertise are generally more familiar with product choices in the market [54,55]. When the customer’s perceived supplier’s professional competence is high, the customer believes that the supplier has the ability to execute the order and believes that they will be able to obtain the right product in a timely manner [20], which will increase customer satisfaction and trust, thereby improving the quality of the relationship [56], and reducing risk perception and price elasticity so that customers can be assured in continuing their transactions [54,57], thus promoting long-term transactions between customers and merchants [21,58].

Agricultural resources, such as pesticides, seeds, and fertilizers, are the basis for increasing agricultural production, quality, and safety of agricultural products and, in turn, increasing farmers’ income. Their high aftereffects have a crucial impact on agricultural production. However, agricultural resources are sensitive to different land types, weather, growing seasons, and time points. If seeds and pesticides cannot be selected to meet the requirements of solar terms and adapt to land conditions, this will lead to weak drought resistance, weak wind resistance, fertilizer inefficiency, and poor efficacy, and, in turn, a reduction in production. As competition is increasingly intense, the brands and prices of products supplied by agricultural retailers in the same village are very often the same, thus the economic benefits brought by the correct guidance of purchasing agricultural resources are prominent. Effective guidance will benefit farmers, making them believe that the resources introduced by competent retailers are the right products for them to purchase [59], thus naturally leading to repeated purchase intention. Therefore, it is proposed that, when all else is equal:

**Hypothesis 2** **(H2).** *The professional competence of agricultural sales staff positively affects farmers’ repeated purchase intention*.

### 3.3. Personal Relationship, Professional Competence, and Repeated Purchase Intention

Hayto classifies personal relationships in the context of inter-firm business relationships into three types, i.e., highly private relationships, strict business relationships, and business friend relationships [60]. The difference between the three relationships is the strength of the emotional elements in the relationship. Among them, a “business friend relationship” that mixes two components of a business transaction and friendship is consistent with Huang’s definition of a “mixed relationship” [61]. The acquaintance relationship between a farmer and an agricultural salesperson is a typical “business friend relationship”. Zhang et al. claim that although the development of friendship in pure business transactions is beneficial to the performance of business relationships, the relationship between business friends contains two conflicting orientations, i.e., interest orientation and emotional orientation, which will make friends’ business relationships face tremendous tension and conflict and have a negative impact on business performance [62].

In the process of rural urbanization and industrialization, the mobility of rural personnel has increased. Villages are changing from the traditionally closed “rural society” to the semi-closed “post-rural” society [14]. Villages are interacting more frequently with people and receiving more information from outside of the traditionally closed world. The convenience of receiving information about the brands, quality, efficacy, and price of agricultural resources, and knowing more suppliers makes the farmers think more rationally. Thus, a conflict between the “human relationship” principle in traditional rural society and the “rational” principle in modern rural society is seen [63]. Therefore, while in the traditionally closed rural society, emotions and personal relationships dominate life and business, in the transition process, sensibility and rationality coexist in farmers’ thinking [16,19]. The two ways of thinking conflict in purchasing decisions and behaviors, thereby reducing the stability of the repeated purchase intention of farmers. This phenomenon falls within the categorization of a “suppressing” relationship between corporate resources in achieving better performance of the firm [64]. Therefore, it is proposed that, when all else is equal:

**Hypothesis 3** **(H3).** *The interaction between personal relationship and professional competence negatively affects farmers’ repeated purchase intention*.

In addition, in order to further ensure the purity of the research model, the irreplaceability of agricultural retail stores and future interaction expectations are adopted here as control variables. The irreplaceability of agricultural retail stores refers to the situation that, in a relatively closed rural environment, due to transportation and geographical difficulties, farmers have only a limited choice of agricultural resources retail stores, and this situation forces farmers to repeat their purchase from certain agricultural retailers, and does not really reflect their subjective willingness [65]. The personal relationship in the conceptual model of this study is a kind of active emotionally subjective will. Therefore, in order to eliminate the passive dependence factor and study the farmers’ active intention more directly, the irreplaceability of agricultural retail stores was adopted here as a control variable [66].

Future interaction expectation refers to the buyer’s willingness to engage with the seller in the future, focusing on the willingness of the buyer to continue interacting with the seller who has provided purchasing services for him/her. According to the definition, future interactions are expected to focus more on communication and interaction, and do not necessarily involve the intention to repurchase. Repeated purchase intention is a predictor of repeated purchase behavior. It emphasizes the intention to repurchase and the ability to take action, not only on communication or other interactions. Therefore, to remove the future interaction components of pure communication and interactions other than to repurchase, and to accurately examine the model, future interaction expectations was chosen as a control variable as well.

**Figure 1:**
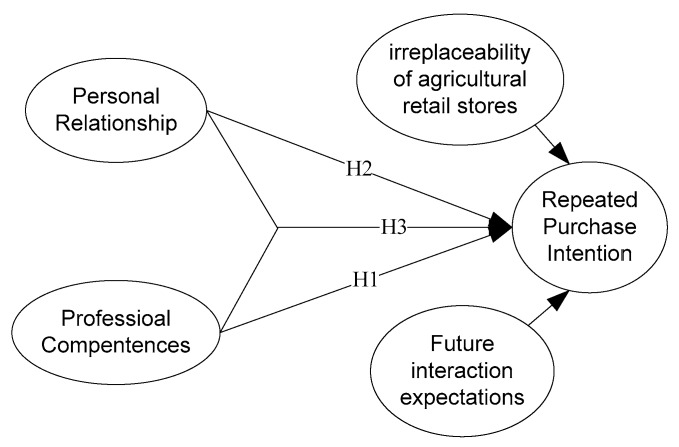
Theoretical model.

Therefore, the theoretical model of this study is shown in Figure 1.

## 4. Research Methods

This study used a questionnaire and hierarchical regression method to examine the theoretical model and test the research hypotheses. To ensure the robustness of the results, the model was then tested again by structural equation modelling.

### 4.1. The Development of the Questionnaire

To ensure the reliability and validity of the questionnaire, the questions and items assessed were developed based on extant research. For the scales quoted from the foreign literature, a two-way English–Chinese–English translation was used to ensure the accuracy and equivalence. The problems found in the translation process were discussed by the team members, and corresponding solutions were formulated and implemented. The preliminary translation completed by the research team was checked by translation experts in this field, and necessary corrections were made accordingly. For the tested scales developed in the domestic literature, these were fine-tuned according to the survey situation in the rural area, so that the interviewees could accurately understand the content of the questionnaire. A Likert 7-level scale was used in the questionnaire, where 1 means disagree completely and 7 means fully agree.

The original questionnaire was then modified according to the research context of this study, and further modified by a pilot study. This was done to not only ensure the quality of the questionnaire but also enable a comparison of the results of this and previous studies. Considering the research context of this study, the questions were carefully phrased to focus on agricultural resource purchasing; the phrases adopted in the questions were designed to meet the educational background of the farmers, so that misunderstanding or confusion would be largely reduced. In addition, the way that the questions were asked was carefully designed to ensure objectivity and neutrality, and to avoid eliciting any unhappy feelings in the farmers.

In order to test whether the questionnaire items were in line with the rural reality and were easy for farmers to understand, a pilot study was conducted after the design of the questionnaire was completed. A village in Tongbai County, Nanyang City, Henan Province of China was randomly selected as the research object of the pilot study. Investigators in the pilot study were postgraduate students from the author’s university. They were trained to understand the purpose and techniques of the survey. In order to ensure that respondents filled in the questionnaire truthfully and accurately, the purpose and meaning of the items were explained to them, and confidentiality was also guaranteed before they started to answer the questions. After the questionnaire was completed, a short interview was conducted to collect feedback and suggestions from the respondents on the questionnaire, and a record was made.

A total of 80 questionnaires were distributed, and 75 valid questionnaires were returned. The recovery rate was 93.75%. After the questionnaires were retrieved, the data were entered into the database, and then analyzed using SPSS 20.0 (IBM, Armonk, New York, NY, United States). The reliability and validity of the questionnaire was assessed. Based on the results of the analysis and the feedback from the interviewees during the survey, the preliminary questionnaire was modified. In order to ensure the reliability of the data, the data collected by the pilot study were not used in the following formal analysis.

### 4.2. Variables

The measurement of personal relationships was based on Wang and Chao [29] and Liu [7]. This measure has 6 items in total, of which the factor loading of the fourth item is low, thus this was eliminated from the analysis. Thus, the final items were the farms believing that salespersons are friends, their willingness to help each other on non-work issues, talking about personal issues when dealing with salespersons at agricultural retail stores, keeping in touch with each other even after the sale and purchase, and believing the salesperson is a member of their inner circle.

The measurement of professional competence was based on Doney et al. [67], which involves a total of 4 items, including items, such as an understanding of products, knowledgeable of the markets, providing solutions to help farmers, and recommending alternative products to meet farmers’ needs.

The measurement of repeated purchase intention was based on Guenzi and Georges [68], with a total of 3 items. The items range from more dealings with retail stores in the future, purchasing new agricultural products and services from old retail stores, and buying more products and services from the old stores.

The control variable “irreplaceability of the agricultural retailer” was measured by the scales developed by Yilmaz et al. [66], which includes items, such as the switching costs of changing agricultural retailers, the difficulty in finding an alternative retailer, and the difficulty in switching to another retailer.

The control variable “future interaction expectation” was measured based on the scale developed by Doney et al. [67]. The items included the certainty of purchasing agricultural resources from the same agricultural retailers in the next one and three years, respectively (see Table 1).

### 4.3. Sampling and Data

A stratified sampling method was used in the selection of survey objects to ensure the effectiveness of the survey. The objects were sampled from three levels, i.e., provinces, cities, and counties. Regarding the selection of provinces, agriculturally developed provinces were selected randomly from the eastern, central, and western areas of China. As a result, Hebei Province was selected from the eastern region, Henan Province was selected form the central region, and Guizhou Province was selected from the western region. In addition, given the significance of agriculture to the economy of the western region, in order to make the study more thorough, the Ningxia Autonomous Region was selected from the western region as well.

The agricultural and geographical conditions of these provinces are as follows. Hebei Province is China’s major producing area of grain, cotton, and edible oil, and contains the country’s 13 commercial grain production bases. Most of its area is located in the North China Plain, with rich arable land and good sunshine. Henan Province is an important production base of high-quality agricultural products in the country, located in the middle and lower reaches of the Yellow River in central China. It enjoys a humid climate, abundant rainfall, and long sunshine hours. The province produces wheat, corn, vegetables, fruits, tea, and herbs. Guizhou has a mild humid climate, rich in heat, and enjoys good agricultural development conditions. The province mainly produces various coarse grain, fruits, tobacco, tea, and vegetables. Ningxia is a plain region in the mid-north of China. It enjoys a unique agricultural foundation, long sunshine hours, high effective accumulated temperature, sufficient precipitation, and long frost-free period. The province mainly produces potatoes, wheat, corn, medlar, and liquorice.

At the second level of the selection, 11 cities were randomly selected from the four provinces: Zhoukou, Luohe, Nanyang, Jiaozuo, Xinzheng, and Jiyuan from Henan Province; Baoding and Hengshui from Hebei Province; Zunyi and Liupanshui from Guizhou Province; and Yinchuan from Ningxia Autonomous Region. Furthermore, 11 counties with strong geographical advantages in agricultural production were selected from the 11 cities. Finally, some representative villages and farmer households were selected from these counties. 

The research object of this study was farmers in each household who were mainly engaged in agricultural production and planting, and had experience in purchasing agricultural resources. These farmers selected covered four categories, i.e., normal farmers, farming experts, village cadres, and agricultural technicians. Normal farmers generally have a low level of education and limited knowledge. When they are purchasing agricultural products, they typically follow most people’s choice, or choose the brand or type of agricultural product based on past experience. The farming experts have a relatively higher level of education, a wider range of knowledge, long planting history, rich experience, a large planting area, and are willing to actively try new products. Their choices often affect the selection of agricultural products by farmers nearby. Farming experts generally make their own decisions when purchasing agricultural products based on their expertise. Village cadres are more authoritative in the countryside; they are often better educated, not necessarily experts in farming, but well informed by a variety of information and interact with more people from outside of the villages. Agricultural technicians specialize in agricultural planting and agricultural product promotion. They have their own professional opinions when purchasing agricultural resources and selecting agricultural resources retailers. A sample profile is shown in Table 2.

Again, the investigators of the formal questionnaire were mainly graduate and undergraduate students from the author’s university. They were purposely selected from farming families, as they were familiar with rural production and life, and were able to communicate with and obtain information from farmers in a way that the latter understood easily. Before the survey started, they were given special training on the questionnaire survey process, questions that needed to be addressed in the survey, techniques, and precautions. The survey was then conducted during the summer holiday from July to September 2016. The questionnaire was distributed and collected by door-to-door visits. A total of 605 rural families were visited, and a person from each family who was familiar with agricultural production and operation was chosen as the respondent. In order to avoid any misunderstanding of the survey questions by the respondents, the questions were explained one by one to them. The questionnaire was then filled out based on the respondent’s answers.

After the survey was completed, two members of the research team entered the data into two databases at the same time to ensure the accuracy of the data input. The two databases were then compared. If the data in the two databases were found to be different, the original questionnaire was checked again to correct the mistakes in the databases. The process was continued until the data was completely consistent. Finally, a part of the questionnaire data was randomly selected and rechecked to ensure that the data input was complete and correct.

A total of 605 questionnaires were collected in this survey, of which 27 were invalid, thus a total of 578 valid answers were used for the formal analysis. The effective recovery rate of the questionnaire was 95.5%. Invalid questionnaires were mainly due to incomplete answers of the questions. Of the valid questionnaires recovered, 99 were collected in the eastern region, 340 were from the central region, and 139 from the western region, accounting, respectively, for 17.13%, 58.82%, and 24.05% of the total sample. The survey data were analyzed and processed using SPSS20.0.

## 5. Results

### 5.1. Common Method Variance

Harman’s single factor test was used here to examine the common method variance of the data. All the items in the questionnaire were put together for unrotated factor analysis, and released a total of five principal component factors, with the first factor having a variance explanatory power of 26.88%, which is lower than the threshold standard of 40%. Thus, the common method variance was not significant and did not affect the research conclusions of this study.

### 5.2. Reliability

This study used internal consistency as the reliability measurement index, and measured the structural reliability of the questionnaire by Cronbach’s α coefficient. SPSS 20.0 was used for the reliability analysis. The reliability coefficient of the total scale reached 0.825, and the reliability of the subscales reached above 0.6, showing good internal consistency.

In order to further assess the internal consistency of the scale, the combined reliability value (CR) of each variable was analyzed. The results showed that the CR value of each variable is greater than the standard threshold of 0.7, and is even close to or higher than the standard of 0.8. Cronbach’s α and CR values showed that the variables of this questionnaire have fairly good reliability and consistency, and the stability is also very good.

### 5.3. Validity

Sample sufficiency and sphericity test results showed that the Kaiser-Meyer-Olkin (KMO) values of personal relationship, professional competence, and repeated purchase intention are 0.756, 0.718, and 0.669, respectively, all of which are greater than the standard of 0.5. The statistical values of Bartlett’s sphericity test were also significant. Therefore, the samples of this study met the criteria for exploratory factor analysis.

Structural validity is divided into convergence validity and discriminant validity. In the structural validity test, the factor analysis results of the five variables of personal relationship, professional competence, repeated purchase intention, irreplaceability of agricultural retail stores, and future interaction expectations are shown in Table 1. The factor loadings of each variable are greater than 0.6 and much larger than the standard of 0.4. The average variance extraction value (AVE) of all factors exceeds the threshold value of 0.5 proposed by Fornell and Larcker [69], indicating that the scale has good convergence validity. In addition, as shown in Table 3, the square root of each variable’s AVE is greater than the correlation coefficients of it with other variables, which meets the requirements of the discriminant validity test. Therefore, the questionnaire has good structural validity.

### 5.4. Correlations Analysis

Pearson coefficients were used in this study for correlation analysis of the variables. Table 3 shows the mean, standard deviation, square root of AVE, and correlation coefficient of each variable. The sample data presents a normal distribution and can be analyzed further.

### 5.5. Results of Regression Analysis

This study used hierarchical regression and SPSS 20.0 software to analyze the data and test the hypotheses. The hierarchical regression analysis of this study included four models as shown in Table 4. Model 1 represents the regression between control variables and repeated purchase intention. Model 2 shows the regression of personal relationship on repeated purchase intention. Model 3 represents the regression of professional competence on repeated purchase intention. Model 5 shows the regression of the interaction between personal relationships and professional competence on repeated purchase intention. In order to minimize the multicollinearity problem between interaction variables, the related variables were averaged before regression analysis.

Model 4 in Table 4 shows the basic effects of the two independent variables on repeated purchase intention. The model fits the data well. Personal relationship (β = 0.249, *p* < 0.001) and professional competence (β = 0.281, *p* < 0.01) all have significantly positive effects on repeated purchase intention, indicating that both personal relationship and professional competence promote repeated purchase intention. Therefore, hypothesis 1 and 2 are supported.

In addition, while both professional competence and personal relationship have a positive effect on repeated purchase intention, with significance at the 0.001 level, professional competence’ influence is slightly higher than that of personal relationship (β = 0.281 vs. β = 0.249), indicating that professional competence is gaining ground in farmers’ purchasing decisions.

Model 6 in Table 4 shows the whole model. When the interaction variable is included, the model also fits the data well. The interaction between personal relationship and professional competence has a negative effect on repeated purchase intention. The coefficient β = −0.088 is significant at the 0.01 level, indicating that the interaction between personal relationship and professional competence reduced the repeated purchase intention. Therefore, hypothesis 3 is supported as well.

### 5.6. Robustness Test

To ensure the robustness of the results, a structural equation modeling (SEM) was adopted here to examine the model and hypotheses as well. AMOS 22.0 (IBM, Armonk, New York, NY, United States) was used to conduct the analysis. Following Kenny and Judd [70], the interaction between personal relationship and professional competence was included in the basic theoretical model as an independent variable. This variable is also a latent variable, and its indicators are the cross-products of the values of the items of personal relationship and professional competence. To minimize the possibility of multicollinearity, the indicators of the independent variables were centralized before the calculation of the cross-products and inputted in the SEM. The result was a group of 20 indicators of the interaction variable.

**Figure 2:**
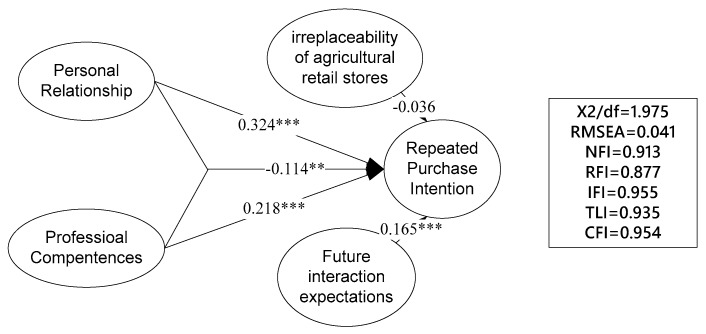
The results of structural equation modeling. Note: *** *p* < 0.01, ** *p* < 0.05.

The three independent variables, i.e., personal relationship, professional competence, and their interaction, were then included in the SEM with the dependent variable, repeated purchase intention, and the two control variables, i.e., the irreplaceability of agricultural retail stores and future interaction expectations. The results show again that the model fits the data well (see Figure 2). Personal relationship has a positive impact on repeated purchase intention (β = 0.324, *p* < 0.001), thus hypothesis 1 is supported. Professional competence positively affects repeated purchase intention (β = 0.218, *p* < 0.001), thus hypothesis 2 is supported. The interaction between personal relationship and professional competences negatively affects the repeated purchase intention of farmers (β = −0.114, *p* < 0.01), thus hypothesis 3 is supported as well. The above results show the strong robustness of the theoretical model.

## 6. Discussion

### 6.1. Theoretical Contributions

Extant studies have shown that interpersonal relationships are closely related to repeated purchase intention [9,63,65]. This study separates personal relationships from interpersonal relationships and defines it as an emotional friend relationship other than a working relationship, and examines its effect on repeated purchase intention. The results show that a personal relationship has a significant positive impact on repeated purchase intention. Due to human emotions, people tend to contact others with better personal relationships, and to provide help to their “own people” and gain mutual trust and support from them. The results of this research clarify the role played by the emotional component of an interpersonal relationship, i.e., personal relationship, in consumers’ purchasing decisions and behaviors. Therefore, compared with extant research, it explores a step deeper, and enables the role played by interpersonal relationships in marketing to be understood in the depth and detail that it deserves.

Previous research has shown that professional competence is closely related to repeated purchase intention [59,67]. The results of this study show that professional competence has a significant positive impact on repeated purchase intention in the agricultural resources market. In this market, the purchase of agricultural resources requires certain expertise, and the absence of rural agricultural technicians and the relatively low level of education of farmers generally lead to the irreplaceable role of agricultural retailers, where they use their professional competence to guide the purchasing decisions of farmers. When choices involve benefits, the rational side of the actor’s thinking will conduct an objective analysis, and that will influence their decision-making and behavior. The findings of this study complement management research on professional competence in marketing, and specifically, the relationship between professional competence and repeated purchase intention.

The results of this study also show that professional competence and personal relationship have similar positive effects on repeated purchase intention. The influence of professional competence is even slightly stronger than that of personal relationships. This result indicates that farmers’ perceptual and rational thinking both play a role in their purchasing decisions. Additionally, the tradition of dealing with others based on emotions and perceptions is increasingly replaced by a new style of mixed rationality and emotion. This finding is consistent with the conclusion drawn in sociology that the Chinese rural society is moving from a perceptual to a rational society, and the current stage is characterized by the coexistence of perceptual and rational social relations. This change is caused by an ongoing process of urbanization and industrialization of rural society. The mobility of rural personnel is increasing, villages are changing from a traditionally closed “rural society” to a semi-closed “post-rural” society [14], and farmers’ perceptions and behavior are changing from a state of high limitations [15] to having more freedom and choices [18].

The extant research has not explored the effect of the interaction between personal relationships and professional competence on repeated purchase intention. The results of this study show that the former has a significantly negative impact on the latter, i.e., the two variables suppress each other in influencing repeated purchase intention [64]. The reason for this result is that farmers face a dilemma in choosing between emotion and financial interest when they make purchase decisions. This dilemma is caused by the conflict between their increasingly rational mindset and traditional perceptual thinking. This dilemma leads to farmers’ hesitation in making choices, and the final decision is a result of the fighting between the two types of thinking, which is thus full of accidents. This, in turn, leads to uncertainty in repeated purchase intention. This is one of the first studies that has investigated the interaction between personal relationships and professional competence on repeated purchase intention. It reveals an interesting scenario that might be found in a transitional market, such as the Chinese agricultural market, and this scenario is caused by the transformation of rural society.

### 6.2. Management Implications

The results of this study have some important implications for practice. Firstly, given the significant positive impact of personal relationships on repeated purchase intention, agricultural retailers need to encourage their sales staff to cultivate personal relationships with farmers so that retailers can establish and maintain customers’ loyalty, ensure a stable customer base, and steadily enlarge their market share. However, it should be emphasized that the personal relationship is more focused on emotions than on other human factors, face, or instrumentality. Therefore, establishing a personal relationship with farmers must be based on true feelings, and aim to become the “own people” of farmers.

In addition, the results of this study show that professional competence has a significant positive effect on repeated purchase intention, thus improving the salesperson’s professional competence is critical to agricultural retailers’ success. Agricultural salespersons should be able to analyze the specific situation of farmers’ production and operation, and recommend suitable agricultural resources based on their understanding of the products and market. In order to do so, they may regularly visit professional institutions, such as seed stations and cooperatives; learn about the latest development of the products; detect problems in farmers’ production and operation in a timely manner; use the knowledge they have to guide the farmers to make the right choices; and help them to seek support from professional institutions if necessary.

Furthermore, as rural society is in a transitional process, agricultural retailers have to understand the dilemma faced by farmers when they are making purchasing decisions. The uncertainty caused by the dilemma may last for a long time, but the tendency of changing from emotional thinking to rational thinking is clear. Thus, agricultural retailers need to understand the importance of professional competence is increasing, and future competition in the market is very likely based on the professional service provided to their customers. Therefore, the strategy here for them may be keeping good personal relationships with farmers while also investing more in improving the professional competences of their salespersons.

Finally, in addition to agricultural retailers, the results of this study will also be useful in practice for agriculture educators, business development educators, rural sociologists, and extension persons working with farmers worldwide.

### 6.3. Limitations and Further Research

This study was conducted in a context of the transition period of rural society, therefore, all arguments and findings might only be applicable to the context of the industry and country studied, i.e., China, and it caution must be used when applying the research results to other situations. However, given the similar characteristics of rural societies in many countries, i.e., the importance of personal relationships, the undergoing urbanization and industrialization (although the speed of this process is different), and the fact that the educational level of farmers is usually lower than urban citizens in most countries, it is expected that the interaction between personal relationships and professional competence in agricultural marketing in countries other than China would present somewhat similar characteristics. Therefore, future studies might look into the relationship between these two variables in other cultural backgrounds. Thus, a deeper understanding of the model and the universality of the results may be gained.

In addition, due to the limitations of the research conditions, the data used in this study were based on a cross-sectional study design. Although the hierarchical regression model and the structural equation model were used to verify the causality between the variables, caution should be used in interpreting the results. Further study may adopt a longitudinal research design, which will be more conducive to discovering changes in the time span.

Finally, from the perspective of perceptions and rationality, this study selected two independent variables, i.e., personal relationship and professional competence, and examined their relationship with repeated purchase intention, and the influencing mechanism. The perceptual perspective could also be based on factors, such as trust, sentiment, and face, and the rational perspective could also be based on the quality of the brand and image of the retailers. Therefore, further research could involve more factors to enrich the content of the two perspectives, and improve the universality and robustness of the conclusions.

## 7. Conclusions

Based on social exchange, rational choice, and perceptual choice theory, this study proposed a theoretical model of the impact of personal relationships and professional competence on repeated purchase intention in the agricultural resources market. Adopting a questionnaire method and hierarchical regression analysis, this study found that both personal relationships and professional competence positively affect repeated purchase intention, but the interaction between the two negatively affects repeated purchase intention. These results reveal the importance of personal relationships and professional competence on the repeated purchase intention of farmers in the agricultural resources market, and the uncertainty caused by the conflict between emotional and rational thinking in farmers’ mindset in a special transitional period of rural society. These results contribute to the marketing theory by more deeply investigating the effect of emotional aspects of interpersonal relationships on repeated purchase intention, by assessing the role played by professional competence in marketing in a special context of agricultural retailing, and by examining, for the first time, the effect of the interaction between personal relationships and professional competence on repeated purchase intention. The results also have important implications for practice.

## Figures and Tables

**Table 1:** Variables and scales.

Scales	Factor Loadings
**Personal relationship (CR = 0.837, AVE = 0.507, Cronbach’s α = 0.752)**	
When local farmers interact with sales staff at agricultural retail stores, think the other party is a friend of himself	0.725
Local farmers are also willing to help each other on non-work issues when dealing with sales staff at agricultural retail stores	0.712
Local farmers often talk about personal issues when dealing with sales staff in agricultural retail stores	0.627
When local farmers interact with the sales staff of agricultural retail stores, they will keep in touch with each other even after the current sale and purchase relationship ends.	0.736
Local farmers think of each other as a member of inner circle of people when dealing with sales staff at agricultural retail stores	0.688
**Professional competence (CR = 0.845, AVE = 0.581, Cronbach’s α = 0.754)**	
Agricultural salesman is knowledgeable in his field	0.830
Agricultural salespersons know the agricultural market very well	0.850
Agricultural sales staff can provide some solutions to help us improve agricultural production	0.696
Agricultural sales staff can recommend alternative products that meet the requirements (e.g., new agricultural products such as pesticides, fertilizers, seeds, etc.)	0.653
**Irreplaceability of agricultural retailer (CR = 0.867, AVE = 0.686, Cronbach’s α =0.767)**	
If the local farmer no longer chooses the agricultural retail store that he frequents, but switches to another one, he can easily make up for the loss of income (R)	0.748
For local farmers, it is easy to find an alternative agricultural retail store (R)	0.900
If local farmers want, it can be quite easy to switch to another agricultural retail store (R)	0.829
**Future interaction expectation (CR = 0.933, AVE = 0.874, Cronbach’s α = 0.875)**	
In the next three years, I will buy agricultural products (agrochemicals, seeds, fertilizers) from the same agricultural retail stores.	0.935
Next year I will buy agricultural products (agrochemicals, seeds, fertilizers) from the agricultural retail stores I visit often	0.935
**Repeated purchase intention (CR = 0.878, AVE = 0.707, Cronbach’s α = 0.789)**	
Local farmers will have more business dealings with agricultural retail stores in the future	0.763
Local farmers buy new agricultural products (pesticides, fertilizers, seeds) or new services from the same agricultural retail stores	0.881
Local farmers will buy more agricultural products (pesticides, fertilizers, seeds) or services from the same agricultural retail stores	0.873

Note: CR, composite reliability; AVE, average variance extracted.

**Table 2:** Sample profile (%).

**Gender**	
Male	66.78
Female	33.22
**Farming Experience**	
<10 years	23.9
10–30 years	38
30 years <	38.1
**Education**	
Less literacy	10.38
Elementary school	31.66
Junior high school	45.67
High school/technical school	11.07
Diploma and above	1.21
**Agri. Income/Total Income**	
0–25%	50.9
26–50%	27.8
51–100%	21.3
**Age**	
<36	10.38
37–46	26.12
47–54	27.85
55–65	23.7
**Family Income (Yuan)**	
<10,000	25.78
10,000–30,000	47.58
30,000–60,000	19.55
60,000–90,000	3.8
>90,000	3.29

**Table 3:** Correlation coefficient between statistical description and each research variable.

Variables	Means	S.D.	1	2		3	4	
1 RPI	5.641	0.996	(0.841)					
2 PR	5.012	1.088	0.395 **	(0.712)				
3 PC	5.494	1.009	0.421 **	0.316 **		(0.762)		
4 IR	3.364	1.537	−0.123 **	−0.080		−0.167 **	(0.828)	
5 EI	5.488	1.232	0.369 **	0.216 **		0.225 **	−0.063	(0.935)

Note: RPI: Repeated purchase intention; PR: Personal relationship; PC: Professional competence; IR: Irreplaceability of retailers; EI: Expected interaction. *N* = 578; The value in parentheses on the diagonal is the square root of the average variation extraction (AVE). The off-diagonal is the correlation coefficient of each variable, ** *p* < 0.01.

**Table 4:** Hierarchical regression.

Independent Variables	Repeated Purchase Intention					
	Mold 1	Mold 2	Mold 3	Mold 4	Mold 5	Mold 6
IR	−0.100 **	−0.81 **	−0.047	−0.043	−0.089 **	−0.036
EI	0.293 ***	0.292 ***	0.287 ***	0.246 ***	0.353 ***	0.244 ***
PR		0.325 ***		0.249 ***		0.254 ***
PC			0.348 ***	0.281 ***		0.265 ***
PR × PC					−0.130 **	−0.088 **
R	0.496	0.572	0.508	0.560	0.403	0.567
R2	0.246	0.327	0.258	0.314	0.162	0.321
ΔR2	0.242	0.322	0.255	0.309	0.158	0.315
F	49.198	62.145	66.663	65.218	36.878	53.904

Note: RPI: Repeated purchase intention; PR: Personal relationship; PC: Professional competence; IR: Irreplaceability of retailers; EI: Expected interaction. *** *p* < 0.01, ** *p* < 0.05.
